# Imprint cytology: a useful screening test for diagnosis of *Helicobacter pylori* in resource poor settings

**DOI:** 10.1186/s13104-018-3592-2

**Published:** 2018-07-16

**Authors:** Piyumali Sandareka Arachchi, Manjula Manoji Weerasekera, Bimalka Seneviratne, Deepaka Weerasekera, Neluka Fernando, Chinthika Prabhashinie Gunasekara

**Affiliations:** 1 Department of Microbiology, Faculty of Medical Sciences, University of Sri Jayewardenepura, Gangodawila, Nugegoda, Sri Lanka; 2 Department of Pathology, Faculty of Medical Sciences, University of Sri Jayewardenepura, Gangodawila, Nugegoda, Sri Lanka; 3 Department of Surgery, Faculty of Medical Sciences, University of Sri Jayewardenepura, Gangodawila, Nugegoda, Sri Lanka

**Keywords:** *Helicobacter pylori*, Diagnosis, Imprint cytology, Toluidine blue, Giemsa

## Abstract

**Objective:**

The study aimed to compare the usefulness of two staining methods for imprint cytology for diagnosis of *Helicobacter pylori* infection. Gastric biopsy specimens (from dyspeptic patients attending routine upper gastrointestinal endoscopy) were placed on glass slides to obtain imprints. The imprints were stained with Toluidine blue and Giemsa stains separately and observed under ×400 magnification using a light microscope. Imprinted biopsies were sectioned and stained with H & E stain and Giemsa stain for histological analysis. Diagnosis of *H. pylori* infection in both imprint and histological sections were confirmed by a consultant pathologist. The sensitivity, specificity, positive predictive value and negative predictive value of each stain were calculated and benchmarked against histological diagnosis.

**Results:**

Of the 55 dyspeptic patients enrolled in the study, 5 were positive for *H. pylori* by Toluidine blue stain and 4 by Giemsa stain. The sensitivity of Toluidine blue stain (57.1%) was higher than Giemsa stain (42.9%) while the specificity of both stains was equal (97.9%). Giemsa stain gave a better discrimination for identification of *H. pylori* bacteria among the mucosal background. Imprint cytology is a rapid, simple and cost effective diagnosis method that can supplement histological diagnosis.

**Electronic supplementary material:**

The online version of this article (10.1186/s13104-018-3592-2) contains supplementary material, which is available to authorized users.

## Introduction

*Helicobacter pylori* diagnosis is a challenging task despite the availability of several diagnostic methods. In Sri Lanka, histological investigations are a main approach for *H. pylori* diagnosis. A drawback of this approach is the long turnaround time incurred due to the laborious preparations involved in specimen processing [1]. Therefore a rapid low cost and simple method to diagnose *H. pylori* infection will enable initiation of treatment immediately.

Imprint cytology enables the visualization of *H. pylori* using a simple staining method. It can further complement histological diagnosis of *H. pylori*. Studies investigating the diagnostic utility of imprint cytology for *H. pylori* report high sensitivity of (83%) and specificity (100%) [2]. Further combination of imprint cytology with histology can improve the accuracy of diagnosis to 100% [3–5]. However currently imprint cytology is rarely used in *H. pylori* diagnosis.

Isolation of *H. pylori* for microbiological diagnosis is difficult due to the fastidious nature of the organisms which require special microaerophilic culture conditions [6, 7]. However imprint cytology will enable microbiologists to identify *H. pylori* based on characteristic morphological appearance in the biopsy smear using commonly available stains thus facilitating an early diagnostic opportunity. This method offers a rapid, cost-effective and simple diagnostic technique which can supplement the routine histological and biopsy urease tests as well as prove to be a valuable method which can be practiced in routine use in the diagnosis of *H. pylori* in developing countries.

## Main text

### Methodology

#### Study population and specimens

Dyspeptic patients attending routine upper gastrointestinal endoscopy at a tertiary care hospital in Sri Lanka were enrolled in the study after obtaining written informed consent. Patients who were less than 18 years of age, patients who were mentally unstable, those who have been on antibiotics for the past month and patients with malignant diseases (e.g., gastric cancer) were excluded from the study population. Fifty five patients were enrolled in the study and the socio-demographic and medical data were collected using an interviewer-administered questionnaire. From each patient, two biopsy specimens were collected for imprints and histology.

#### Specimen processing

The two biopsy specimens were placed on clean glass slides separately and imprints were obtained from each biopsy. After obtaining an imprint, each biopsy was placed in 10% formal saline (Welcome chemicals, Sri Lanka). The imprint slides were air-dried. The biopsy specimens were transported to the Department of Pathology at Faculty of Medical Sciences, University of Sri Jayewardenepura for histopathological investigations.

#### Histopathological investigations

The biopsy specimens were dehydrated using an alcohol gradient, embedded in paraffin wax and sectioned into four micron sections which were placed on clean glass slides. The sections were stained with Hematoxylin (Avondale, England) and Eosin (BDH, England) stain and Giemsa stain (LOBA chemie, India) before being graded according to the updated Sydney system [8] by a consultant pathologist.

#### Imprint cytology of biopsy specimens

After air-drying, one imprint specimen was flooded with 0.5% Toluidine blue stain (Himedia, India) and washed with water after 1 min [9]. The second specimen was placed in a trough containing Giemsa working solution (LOBA chemie, India) and allowed to stain for 15 min before washing with running water [10]. The stained slides were dried and mounted using DPX mounting medium (Merck, India). The slides were observed under ×400 magnification using a light microscope.

### Results and discussion

The *H. pylori* infection was investigated in gastric biopsies taken from 55 dyspeptic patients (Additional file 3: Book 1). Of these patients 34 were male while 21 were female with a median age of 56 (age range from 21–82). Of the 55 dyspeptic patients, 7 were diagnosed as *H. pylori* infected by histology. Five patients (5/55) were diagnosed as *H. pylori*-positive by Toluidine blue stain while 4 (4/55) were diagnosed with Giemsa stain.

**Fig. 1:**
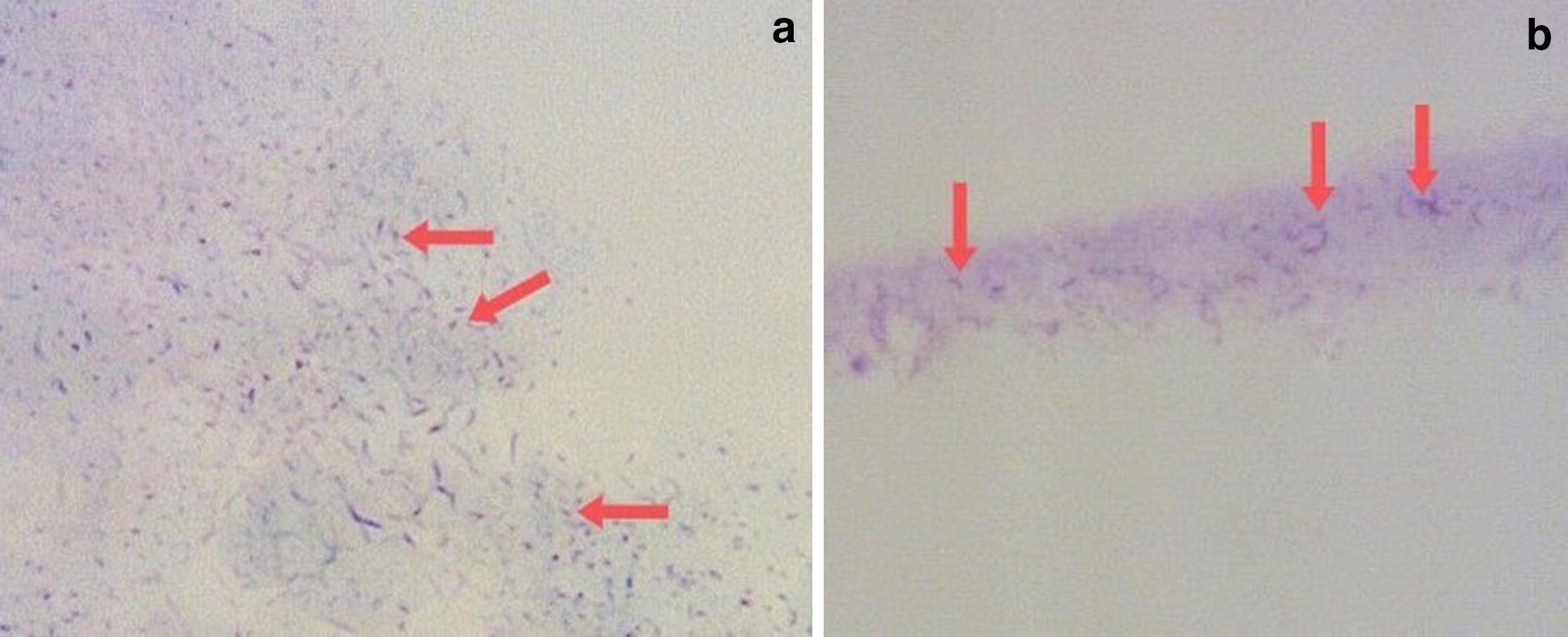
Toluidine blue staining of *H. pylori*-positive specimens with their characteristic curved-spiral rod shape (×400 magnification). **a** Imprint smear specimen with high density *H. pylori*; **b** Low density *H. pylori* in dark blue among mucus stained light blue

**Fig. 2:**
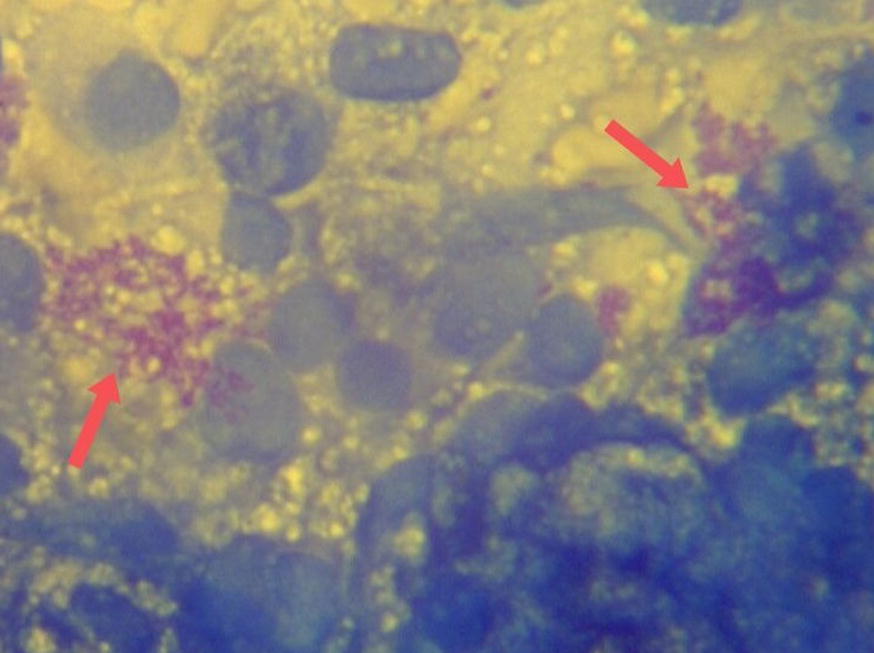
Image of a *H. pylori*-positive imprint slide stained with Giemsa stain (×400 magnification). Epithelial cells are stained in dark blue while *H. pylori* bacteria can be seen in magenta color

Both Toluidine blue and Giemsa staining of the imprints revealed the strongly stained unique morphology of *H. pylori* visible as curved or spiral shaped rods embedded in the gastric mucosa. On comparison, Toluidine blue stained smears revealed the characteristic morphology of *H. pylori.* The meta-chromatic properties of this dye enable the differentiation of *H. pylori* from the mucus and the epithelial cells, where the bacteria stains dark blue against a variably blue background [11]. Toluidine blue staining enabled observation of both high (Fig. 1a) and low density (Fig. 1b) of *H. pylori* in the imprint smears. When slides containing gastric biopsy imprints were stained with Giemsa stain, the epithelial cells on the slide were visualized in blue color while the *H. pylori* bacteria were stained in magenta color which enabled better discrimination of the bacteria compared to Toluidine blue stain (Fig. 2). Giemsa being a multiple stain contains three dyes: methylene blue, Azure B and Eosin which contributes to better discrimination of bacteria from the background. By the Giemsa stain, the nuclear material of both bacteria and cells are stained dark blue to violet and the mucus is stained pale blue [12]. However Toluidine blue was more useful to determine the bacterial density compared to the Giemsa stain.

Considering the histological diagnosis of *H. pylori* as the benchmark, the sensitivity specificity, positive predictive value (PPV) and negative predictive value (NPV) of Toluidine blue stain and Giemsa stain are described in Table 1. Sensitivity of Toluidine blue staining for diagnosis of *H. pylori* infection was 57.1% while the specificity was 97.9%. The PPV and NPV of Toluidine blue staining was 80.0 and 94.0% respectively, when compared with histological diagnosis. Diagnosis of *H. pylori* by Giemsa stain had a sensitivity of 42.9% and a specificity of 97.9% compared with histological diagnosis. The PPV and NPV of Giemsa stain was 75.0 and 92.2% respectively. The sensitivity of Toluidine blue stain was higher than Giemsa stain while both stains had similar specificity.

**Table 1:** Comparison of the two stains used for imprint cytology in the diagnosis of *H. pylori* infection

	Sensitivity (%)	Specificity (%)	Positive predictive value (%)	Negative predictive value (%)	Kappa coefficient
Toluidine blue stain	57.1	97.9	80.0	94.0	0.627
Giemsa stain	42.9	97.9	75.0	92.2	0.499

Of the seven specimens diagnosed as *H. pylori*-positive by histology, four specimens were positive for *H. pylori* by Toluidine blue stain while three were positive by Giemsa stain. Further, of 48 *H. pylori* negative (by histology) biopsy specimens *H. pylori* was identified in one specimen by both staining methods.

Of the seven *H. pylori*-positive specimens, three were diagnosed with high *H. pylori* density and the remaining four with low *H. pylori* density by histology. All three high density specimens were diagnosed as positive by both Toluidine blue and Giemsa stains. One low density specimen was diagnosed as positive by Toluidine blue stain but not by Giemsa stain. Low density specimens being diagnosed as *H. pylori*-negative by both stains may be due to poor transfer of bacteria from the biopsy specimen to the slide which may be a disadvantage of the imprint cytology method.

When considering a single biopsy specimen, imprint cytology was found to be a simple staining method which maximizes the use of this biopsy obtained for histological investigations without affecting the results of histology [4, 13]. Thus the quality of the imprint as well as the integrity of the tissue which was sectioned needs to be high enabling histological review. Therefore during the preparation of imprints care should be taken to ensure that the biopsy specimen is undamaged by gentle handling by rolling rather than pressing hard on the biopsy specimen or dragging the specimen across the slide [13]. As the same biopsy specimen can then be used for histological investigations, use of imprint cytology can maximize the diagnostic utility of the biopsy specimen.

Several reports indicate a high sensitivity and specificity by imprint cytology [5, 14]. However, in this study while imprint cytology was 100% in agreement with high density *H. pylori* containing biopsy histology, the positivity for low density *H. pylori* containing biopsy was poor as has been reported by other study groups [4]. A disadvantage of imprint smear preparation is that the low density specimens may be diagnosed as *H. pylori* negative due to poor transfer of bacteria from the biopsy specimen onto the slide. Further, processing or staining errors may reduce the sensitivity of the imprint cytology diagnosis by giving rise to artifacts in the imprint slide which may give false positive results as can be seen in this study where one specimen *H. pylori*-negative by histology was diagnosed as positive by both imprint stains.

While histology is widely used for diagnosis of *H. pylori* infection a major drawback of this approach is the need for sophisticated equipment and protocols as well as high technical skills of the laboratory personnel during the processing of the biopsy. In contrast imprint cytology can be carried out within 30 min of the collection of biopsy providing a tremendous advantage as therapy can be commenced on the same day before the patient leaves the endoscopy unit [2]. The histological investigations enable both the diagnosis of *H. pylori* infection as well as determination of the histological severity. However, imprint cytology cannot be used to provide information on mucosal inflammation, metaplasia, atrophy and other histological changes but will support the diagnosis of *H pylori*.

Stool antigen test and Realtime PCR are several diagnostic methods that have also been introduced to diagnose the *H. pylori* infection. Both diagnostic methods have a high sensitivity and specificity in *H. pylori* diagnosis and can be carried out as non invasive assays. However, both these methods are expensive when compared with the imprint cytology method as these methods require specific equipment and expensive reagents to carry out the diagnosis.

Further this method is not labor intensive and facilitates rapid interpretation of results compared to 3–5 days needed for the histological report. Both Toluidine blue and Giemsa stains can be prepared beforehand and applied directly to imprint slides. However, the Toluidine blue stain has to be stored at 4–8 °C when not in use while the Giemsa solution can be stored at room temperature. When considering the cost per test, both Toluidine blue stained and Giemsa stained imprint smears incurred a very low cost of approximately Rs. 5.00–10.00 per slide. Therefore using imprint cytology as a screening test before performing histology will not increase the overall diagnostic cost significantly.

### Conclusion

In conclusion, although imprint cytology alone is less sensitive than histology in diagnosis of *H. pylori* infection, it offers a rapid, economical and simple screening method which can supplement histological findings thus increasing the diagnostic sensitivity. Further the biopsy specimen used for imprint smear preparation can subsequently be used for the histological investigations thereby maximizing the diagnostic utility of the biopsy specimen. Increasing the number of specimens taken (from 2 to 5) and taking specimens from different places of the stomach for imprint cytology and histology may increase the accuracy of *H. pylori* diagnosis.

### Limitations

The prevalence of *H. pylori* infection among the study population may have been affected by the use of PPI medication, as PPI use is reported to shift *H. pylori* from antrum to the corpus. The biopsy specimens were collected from the antrum of the patients’ stomach which may have lowered the proportion of *H. pylori* infected patients.

As can be observed in the study, the bacterial density affects the sensitivity of the imprint cytology and specimens with low *H. pylori* density resulted were diagnosed as negative by imprint cytology.

During sample processing and staining, errors can cause the appearance of artifact which may lead to false positive diagnosis.

## Additional files

**Additional file 1: Figure S1.** Image of a *H. pylori*-negative slide stained in Toluidine blue stain (× 400 magnification). Bacilli, stained in dark blue can be observed among the mucus and epithelial cells.

**Additional file 2: Figure S2.** *H. pylori*-negative imprint slide stained in Giemsa stain (x400 magnification).

**Additional file 3: Book 1.** Contains detailed results of all subjects enrolled for the study.

## Abbreviations

NPV: negative predictive value
PPI: proton pump inhibitor
PPV: positive predictive value
