# Supplementary figures and images for: Imprint cytology: a useful screening test for diagnosis of Helicobacter pylori in resource poor settings
Source: BMC Res Notes. 2018 Jul 16;11:481. doi: 10.1186/s13104-018-3592-2 (PMC6048746; doi:10.1186/s13104-018-3592-2)

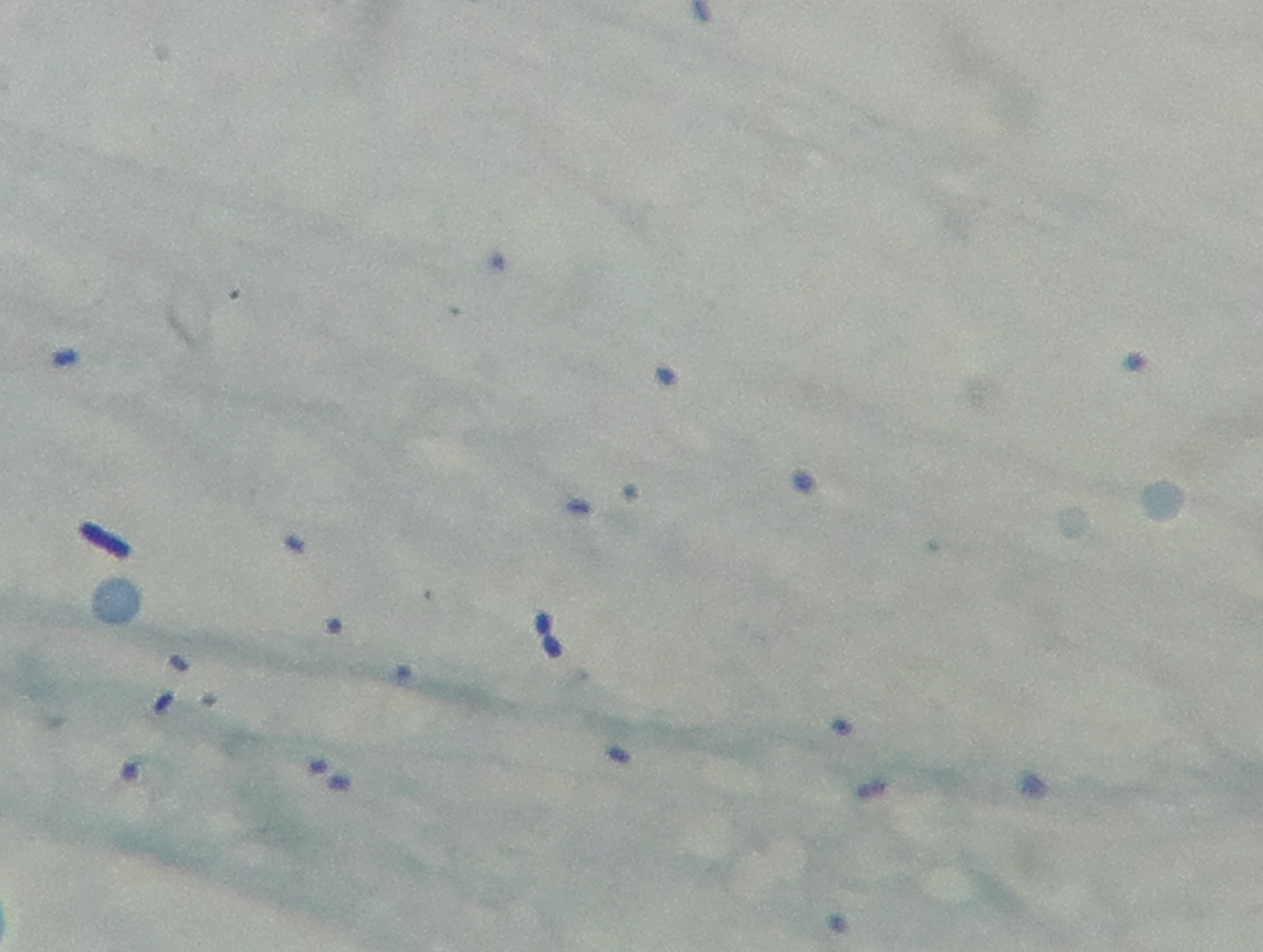

Supplement: Supplementary file 1 — Additional file 1: Figure S1. Image of a H. pylori-negative slide stained in Toluidine blue stain (× 400 magnification). Bacilli, stained in dark blue can be observed among the mucus and epithelial cells. [file 13104_2018_3592_MOESM1_ESM.jpg]

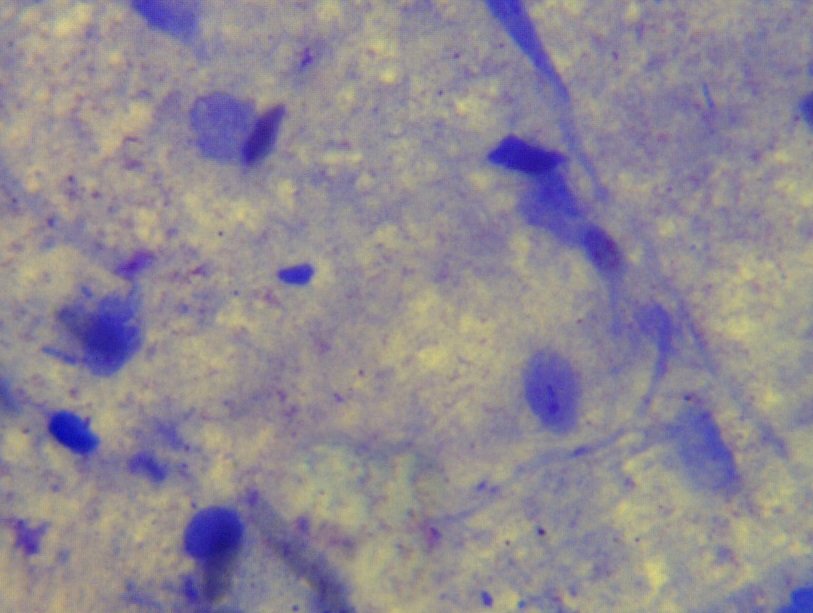

Supplement: Supplementary file 2 — Additional file 2: Figure S2. H. pylori-negative imprint slide stained in Giemsa stain (x400 magnification). [file 13104_2018_3592_MOESM2_ESM.jpg]
